# Enhancing student perspectives of humanism in medicine: reflections from the Kalaupapa service learning project

**DOI:** 10.1186/s12909-016-0664-7

**Published:** 2016-05-09

**Authors:** Winona K. Lee, Chessa C. D. Harris, Kawika A. Mortensen, Linsey M. Long, Jeanelle Sugimoto-Matsuda

**Affiliations:** The University of Hawai’i at Mānoa - John A. Burns School of Medicine, MEB 306H, 651 Ilalo Street, Honolulu, HI 96813 USA; Department of Native Hawaiian Health, John A. Burns School of Medicine, 677 Ala Moana Blvd, Suite1016B, Honolulu, HI 96813 USA; Department of Psychiatry, Research Division, The University of Hawai’i at Mānoa, Suite 301, 677 Ala Moana Blvd, Honolulu, HI 96813 USA

**Keywords:** Service-learning, Diversity, Healthcare workforce, Native Hawaiian, Humanism

## Abstract

**Background:**

Service learning is endorsed by the Liaison Committee on Medical Education (LCME) as an integral part of U.S. medical school curricula for future physicians. Service learning has been shown to help physicians in training rediscover the altruistic reasons for pursuing medicine and has the potential to enhance students’ perspectives of humanism in medicine. The Kalaupapa service learning project is a unique collaboration between disadvantaged post-baccalaureate students with an underserved rural community. This study was conducted to determine whether the Kalaupapa service learning curricula enhanced student perspectives of humanism in medicine at an early stage of their medical training.

**Method:**

Program participants between 2008 and 2014 (*n* = 41) completed written reflections following the conclusion of the service learning project. Four prompts guided student responses. Reflections were thematically analyzed. Once all essays were read, team members compared their findings to condense or expand themes and assess levels of agreement.

**Results:**

Emerging themes of resilience and unity were prominent throughout the student reflections. Students expressed respect and empathy for the patients’ struggles and strengths, as well as those of their peers. The experience also reinforced students’ commitment to service, particularly to populations in rural and underserved communities. Students also gained a deeper understanding of the patient experience and also of themselves as future physicians.

**Conclusion:**

To identify and address underserved and rural patients’ health care needs, training programs must prepare an altruistic health care workforce that embraces the humanistic element of medicine. The Kalaupapa service learning project is a potential curricular model that can be used to enhance students’ awareness and perspectives of humanism in medicine.

## Background

The Liaison Committee on Medical Education (LCME), the accreditation body of U.S. medical schools, has endorsed service learning as an integral part of training future physicians, requiring that it be offered in medical schools nationwide. Service learning, grounded in the pedagogical theories of John Dewey, emphasizes experiential learning outside of the classroom that is then brought back via student reflection and application of new insights and perspectives [1]. Service learning combines academic topics with community service that is mutually beneficial to the student learner and the community involved [2–4]. Many medical students begin their careers with a strong interest in community health and serving the underserved [5]. However, dedicating time to reinforce the humanistic aspects of medicine may be limited when taught within the confines of clinics and hospitals which must focus on the acquisition of necessary biomedical knowledge and procedural skills [5, 6]. Though few question the importance of incorporating humanistic elements of training for future physicians, medical educators are challenged to find a proven method by which humanistic values, perspectives and behaviors can be instilled into trainees [7–10].

Humanism comprises a set of personal convictions about one’s obligations to others, especially others in need [11]. In medicine, humanism is demonstrated by a respectful and compassionate relationship between physicians, other members of the healthcare team, and their patients. It reflects attitudes and behaviors that are sensitive to the values and cultural backgrounds of others. Humanistic health care professionals are intuitively motivated to provide health care services with integrity, excellence, compassion, altruism, respect, and empathy [12]. Prior studies have shown that humanistic medical services can positively impact patient satisfaction, promote trust in their physicians and potentially improve health outcomes [13, 14], reinforcing the long-term benefits of integrating medical humanities into training curricula for future physicians and the importance of incorporating these teaching experiences into medical school programs [15–17]. Although service learning in medical education has been shown to develop participants’ skills related to teamwork, communication, and civic engagement and responsibility, the authors are not aware of published service learning curricula that have specifically examined the impact of service learning on students’ awareness of humanism in medicine [18]. This study describes curricular components of a service learning project that may potentially enhance post-baccalaureate student perspectives of humanism in medicine.

### ‘Imi Ho’ōla post-baccalaureate program

As part of the University of Hawai’i John A. Burns School of Medicine’s (JABSOM) longstanding commitment to diversity, the ‘Imi Ho’ōla (Hawaiian meaning “those who seek to heal”) program is a successful educational pipeline for underrepresented and disadvantaged students to enter medicine. ‘Imi Ho’ōla’s mission is to improve health care for Hawai’i and the Pacific by increasing the number of physicians through a one-year enrichment program. ‘Imi Ho’ōla is a post-baccalaureate curriculum that accepts up to twelve (12) students from economically, socially, and/or educationally disadvantaged backgrounds who possess the potential to succeed in medicine and demonstrate a commitment to serve underserved communities in Hawai’i and the Pacific. Upon completion of the program, students matriculate into the first-year medical class at JABSOM. ‘Imi Ho’ōla is institutionalized and is a program located in the Department of Native Hawaiian Health at JABSOM [19]. Since its inception in 1973, Two hundred and forty-five (245) ‘Imi Ho’ōla alumni have matriculated and graduated from JABSOM.

### History of Kalaupapa

In 1865, the Kingdom of Hawai’i passed the “Act to Prevent the Spread of Leprosy” which gave police and district justices rights to arrest anyone suspected of having leprosy, evaluate their condition and forcibly relocate them to Kalaupapa on the island of Moloka’i. An estimated 8,000 patients were sent to the settlement, permanently separating them from their families and society [20]. At least 90 % of these patients were Native Hawaiian [21]. Patients sent to Kalaupapa dealt with food shortages, lack of medical care and supplies, inadequate housing, loss of personal freedoms, mandatory relocation of children, cultural restrictions, and government negligence [22]. These, combined with lack of a cure, stigmatization due to leprosy and complete loss of personal support systems, made Kalaupapa synonymous with imprisonment and death. A few dedicated individuals, such as Father Damien, advocated for patients’ rights but in general, patients were left to fend for their own survival. In 1941, Dr. Guy Faget developed a cure for leprosy. Subsequently, Hawai’i’s isolation policy was lifted in 1969, giving patients the choice to leave the settlement [20]. Although free to leave, decades of mistreatment and stigma made the patients’ re-entry into society difficult, and many former patients chose to remain in Kalaupapa and share their personal stories from the place that had become their permanent home.

### The Kalaupapa service learning project

The Kalaupapa service learning project formally began in 2008. However, the idea of connecting students with this rural, underserved community of Indigenous patients and residents was conceived by former ‘Imi Ho’ōla Director, Dr. Benjamin Young nearly 40 years ago. In 1976, Dr. Young was sailing on the Hokule’a, as part of the Polynesian Voyaging Society. The ship anchored at Kalaupapa on their way back to Honolulu from Tahiti. It was following that visit, that Dr. Young and Mr. Richard Marks planned the first student huaka‘i (fieldtrip). Mr. Marks was a respected historian, successful businessman and Sheriff. He along with his wife Gloria have hosted students annually since 1977 until his death in 2008. During the visits, students would learn about the community by engaging with patients and residents in the hospital and community centers. Students and hosts would also participate in cultural activities such as fishing and traditional food preparation. Mrs. Marks has continued to work with the program to identify the community service needs and learning activities that the students engage in while in Kalaupapa. The Marks family, as leaders of the community and advocacy group, Ka’Ohana O Kalaupapa (the family of Kalaupapa) emphasized the importance of respecting patients’ experiences and preserving the history of Kalaupapa. In 2008, the program developed and implemented the curriculum that is now known as the Kalaupapa service learning project. Mrs. Marks continues to provide direct input to the project by identifying the student service activities based on community needs. Mrs. Marks also coordinates hospital visits with remaining patients and tours of historical sites. At the end of the visit in Kalaupapa, students host a lu’au (traditional Native Hawaiian celebration) for the Marks family in apprecation of the privilege to spend time with the Kalaupapa community.

The Kalaupapa service learning project is integrated into ‘Imi Ho’ōla’s Scientific Basis of Medicine (SBOM) course. Learning objectives for SBOM are to 1) Explain and give examples of varying global perspectives regarding health and disease; 2) Describe the need for healthcare services in rural and underserved populations; 3) Articulate the roles of health professionals working in the health care setting; 4) Predict the epidemiological and psychosocial impact diseases/health conditions have on society; and 5) Recognize and apply professional behaviors and attitudes as recommended by the Association of American Medical Colleges (AAMC). During the Spring semester, Hansen’s disease is discussed utilizing multiple teaching methods, and the history of Kalaupapa as a settlement for Hansen’s disease patients in Hawaii is used as a case study to promote student understanding of how individuals with widely stigmatized diseases are impacted through cultural, religious, economic, and political perspectives. The Kalaupapa service learning project is considered the capstone of this learning process, as it culminates with a three-day immersive experience in Kalaupapa, Moloka’i. Continued student learning regarding leprosy and its impacts is gained through interaction with the remaining patients at Kalaupapa. The Kalaupapa service learning project is a highlight of the SBOM course. Components of the service learning project are described in Table 1.

**Table 1:** Kalaupapa Service Learning Project - Components and Activities

Component	Activity
Fundraising	Students raise a total of $3,000 to be used to support travel expenses including meals, equipment/tools and host gifts
Coordination and Implementation	Students are responsible for purchasing and planning the service project (i.e. providing meals, equipment/supplies)
Problem-based learning health care problem	Students create learning issues related to Hansen’s disease that are researched and presented individually to the class
Individual student research and thesis paper	Students conduct research relevant to Hansen’s disease utilizing standard texts and journal articles. Using their research, students develop a 10 page thesis paper on the topic
Required reading and written exam on *Holy Man: Father Damien of Molokai* by Gavan Daws	Students complete the required reading and take a written exam to assess their comprehension and critical analysis of the book
Oral Presentation	Students present a 10 min presentation on their thesis paper
Service in Kalaupapa	Students complete a 3 day project that may include cleaning yards, painting, or visiting residents. Students also conduct a luʻau (traditional celebration) and talent night for the hosts
Tour of Kalaupapa	Hosts take students on a tour that highlights contributions of Saint Damien and historical sites of Kalaupapa
Reflection Paper	Students complete a reflection paper on their service learning project experience

## Methods

Data were extracted from student reflection essays that were written in response to the Kalaupapa service learning project. As part of the service learning experience, students were required to submit a written self-inquiry about their experience while at Kalaupapa. Four prompts guided student responses (see Table 2). Thus, this study took a phenomenological approach, which examines the meaning of events through the lens of the people closest to the event or situation [23]. In addition, use of the reflection essays allowed for inclusion of the students’ exact words and voices, as opposed to summaries or second-hand information. Data access and research procedures were approved by the Institutional Review Board of the University of Hawai’i at Mānoa. All available essays from students who participated in classes of 2008–2013 were included in the analysis. The demographic profile of the 41 student participants who completed reflections are described in Table 3. A total of 41 reflection essays were thematically analyzed by the four primary individuals on the research team [24]. Prior to analysis, essays were de-identified by an unbiased source. Personal identifiers such as name, date, class, and instructor were removed from each essay. The essays were assigned an identification number and randomly assigned to the group for analysis. Each essay was read in its entirety by two members of the research team who independently identified emerging themes for each of the four prompts. Allowing codes and themes to emerge from the reflections supported the flexibility that is needed in qualitative research to be grounded in and reflective of the voices of the participants [25]. In addition, validity and reliability were ensured through multiple independent sources evaluating the same data [24]. Team members were instructed to review each essay in four stages: 1) read without highlighting or marking; 2) highlight key words; 3) assign names for themes that describe highlighted key words; and 4) review themes and expand or consolidate. All members worked from a common matrix template to organize their key words and themes for each of the four prompts. Once all essays were read and reviewed independently, team members then came together and compared their findings. This multi-session process was facilitated by a fifth member of the research team. During these group sessions, themes within each prompt were condensed or expanded, based on consensus. After the themes were established, related quotes from the essays that could exemplify the themes were added.

**Table 2:** Student Reflection Prompts

Number	Prompt
1	Based on what you observed and experienced during your time in Kalaupapa, discuss the role of disease and how it has impacted the individual, family, community, and society of Kalaupapa.
2	What did you learn about yourself through your experience at Kalaupapa?
3	What did you learn about others?
4	How could a future trip to Kalaupapa be improved to better facilitate your learning?

**Table 3:** Demographic Profile of Post Participation Student Reflections (*N* = 41)

Descriptor	Frequency	Percent (%)
Gender		
Male	19	26.3
Female	22	53.7
Origin		
Hawaii	33	80.5
Guam	7	17.1
American Samoa	1	2.4
Origin		
Rural	27	65.9
Urban	14	34.1
ESL		
No	12	29.3
Yes	29	70.7
Primary Ethnicity		
Hawaiian	9	22.0
Filipino	12	29.3
Asian (Chinese, Japanese, Korean, Vietnamese)	13	31.7
Chamorro	5	12.2
American Indian	1	2.4
Caucasian	1	2.4
Indigenous		
No	26	63.4
Yes	15	36.6
Age Upon Entry (*N* = 41)		
Min | Mean | Max	22 | 26 | 34	

## Results

The demographic profile of the participants who completed post-participation reflections are summarized in Table 3. Student participants in the Kalaupapa service learning project are from disadvantaged backgrounds and the majority of participants originated from rural communities of Hawai’i and the Pacific (i.e. Guam and American Samoa). Ninety-five (95 %) of the students included in the study are of Asian and Pacific Islander descent and seventy-one (71 %) self-identifiy as having English as a Second Language (ESL).

Question 1. Based on what you observed and experienced during your time in Kalaupapa, discuss the role of disease and how it has impacted the individual, family, community, and society of Kalaupapa.

Question 1 was intended to illicit responses that would support LO1) explain and give examples of varying global perspectives regarding health and disease; LO2) describe the need for healthcare services in rural and underserved populations; and LO4) predict the epidemiological and psychosocial impact diseases/health conditions have on society.

### Sense of loss and resulting isolation

Student reflections described an immense sense of loss experienced by patients with Hansen’s disease due to the forced separation from their ‘ohana (family unit). Adults and children, once diagnosed with the disease, were forcibly taken from their families and sent to Kalaupapa, Moloka’i. This physical and emotional separation led to strong feelings of isolation for the patients sent to Kalaupapa. The isolation experienced by patients was further impacted by the remote geographical location of the settlement. Kalaupapa remains underdeveloped and is characterized by vast open fields, small houses, paved roads, cemeteries, and remnants of its historical sites that symbolize the experiences endured by the patients of Kalaupapa.

“The term “isolation” cannot be truly understood until one experiences it oneself… Families were torn apart. Mothers were unable to raise their children, divorce was sanctioned for those spouses that had the disease, and patients were isolated from the larger community.” (Student Reflection #5)

“Once an individual was diagnosed with Hansen’s disease, they were basically marked as a prisoner and cast away to live in this remote peninsula. Many of their rights were taken away and they had no choice but to make this their new home, while leaving behind loved ones and friends. Many times, individuals were alone and had to battle this separation along with their disease.” (Student Reflection #41)

### Unity

Student reflections also described the unification of the patients and residents of Kalaupapa. The creation of strong relationships between individual patients, among healthcare staff and patients, and the larger Kalaupapa community emerged repeatedly in the reflections. Residents and patients found unity through common health and social challenges, as well as a unifying sense of faith and spirituality.

“After examining the land and the people of Kalaupapa, I noticed that it is a very close-knit community where everyone is like family…The community rather than stigmatized the residents, view these people as their role models…who despite the sufferings, continued to strive forward with a positive outlook on life.” (Student Reflection #32)

### Resilience

The theme of individual resilience, demonstrated by the residents of Kalaupapa, also emerged in student reflections. The shared experience of historical trauma and isolation eventually led to the creation of lasting relationships between individuals, families, and communities. The resilience demonstrated by inflicted patients was strengthened by the presence of spirituality and hope.

“I sensed that Kalaupapa was an incredibly peaceful and spiritual place because it was made up of people who had already had their share of unrest in the past…Their inspiring spirituality that spoke of strength in self, forgiveness towards others, and motivations to keep moving forward no matter the obstacles that stand in the way.” (Student Reflection #34)

Question 2. What did you learn about yourself through your experience at Kalaupapa?

Question 2 gives students the opportunity to reflect on LO5) recognize and apply professional behaviors and attitudes as recommended by the AAMC.

### Awareness of self and others

Students gained self-awareness, recognizing their ability for self-reflection and subsequent personal and professional growth. They grasped that their openness to this process of growth was vital to evaluating one’s own personal strengths and weaknesses. Understanding the communities’ hardships allowed the students to reflect on their own strengths and weaknesses, ultimately enabling them to gain increased empathy toward others and affirmation of their original altruistic motives for entering medicine.

“Through this experience, I am increasingly proud and grateful to have been born and raised in the beautiful and historically rich islands of Hawaii. I am also proud to be a part of a resilient culture, Hawaiian, that was able to bounce back from hardships and discrimination that plagued its path....this experience also reminds me to be appreciative of what I have today, never to take things for granted, and by no means should I be afraid of hard work to reach my goals in life.” (Student Reflection #27)

“It’s easy to read about these accounts but when you are watching them tell their stories, with tears in their eyes, I could not help but feel a degree of pain they felt. I learned that people with disease are more than just the disease they have....when I listened to the residents, I was able to put myself in their shoes and experience a piece of their journey.” (Student Reflection #4)

### Resilience

Students expressed that through increased self-awareness and understanding of relationships with others, they learned how resilient they were. Traits that were repeatedly reflected upon included mental and physical strength, perseverance and fortitude.

“What [the patients shared] was an inspiration and has motivated me that there is nothing in life that I cannot overcome and that as long as I have the will to move forward, I will have the strength to tackle it.” (Student Reflection #32)

### Reinforced future professional goals

Thematic analysis revealed that students reinforced their perspective of future goals, reflected on their motives for entering medicine and affirmed their desire to continue to work toward reaching those goals.

“Through my personal experience at Kalaupapa, I have reaffirmed that I eventually want to serve rural communities that are in dire need of physicians. The experience has taught me that my duty is to those who need me…I have noticed and appreciate the tight-knit relationship between patient and doctor, and those are the types of relationships that I hope to build in the future.” (Student Reflection #1)

Question 3. What did you learn about others?

Question 3 helps students apply their perceptions to concepts associated with LO3) articulate the roles of health professionals working in the health care setting; and LO5) recognize and apply professional behaviors and attitudes as recommended by the AAMC.

### Teamwork

Student reflections described the need to put forth their best effort and work together to achieve the goals of the learning service project. As students experienced success with group tasks and activities, a greater sense of respect, support, and trust of their peers developed.

“I learned that I am part of a very dynamic and altruistic group of students. Everyone has qualities, resources or characteristics that add to the success of our group as a whole.” (Student Reflection #36)

### Fellowship

Student reflections described that the opportunity to work together outside the normal academic environment promoted the breakdown of traditional roles in favor of fellowship among group participants. Student reflections revealed that relaxed social environments advanced bonding and camaraderie among participants. The lu’au (i.e. traditional Hawaiian gathering) and participant talent show offered opportunities for open self-expression and team spirit, fostering a sense of community among the participants.

“It was also nice to get to know the faculty members outside of the academic setting. I was able to realize that they too are very much like us, each having unique characteristics that I would have never known about inside the classroom.” (Student Reflection #35)

“Sharing the experiences on the trip brought all of us closer together, not just as classmates but also as a family.” (Student Reflection #16)

### Future

Many of the student reflections stated that the bonds established during their shared experience at Kalaupapa would continue well past graduation and into their future careers. Students felt that they could rely on their classmates for support and help in both the immediate and distant future. Participants were also able to identify how the experience influences them as future physicians.

“This experience has brought our class closer than we were before and I will always be grateful for that. I will take what I have learned to guide me as I work towards becoming a physician and when I am a physician.” (Student Reflection #26)

Question 4. How could a future trip to Kalaupapa be improved to better facilitate your learning?

Although question 4 did elicit student responses that were concerned with future improvements, the nature of the responses resonated with LO5) recognize and apply professional behaviors and attitudes as recommended by the AAMC.

### Length of stay

The overall consensus of student reflections was that the time at Kalaupapa should be extended. During the service learning project, students became invested in the culture and the people around them. Regardless of time constraints, it was also evident in the reflections that the students’ goals for their careers were reinforced during their time at the settlement.

“An additional suggestion for improvement would be to extend the length of the trip by at least one day, so that there would be either time do additional activities, such as visit the hospital, or more time for each activity. Although we managed to accomplish many things during our short trip, many aspects felt rushed and thus could not be fully appreciated or enjoyed had there been more time.” (Student Reflection #21)

### Level of interaction

It was communicated from students that there was a desire for increased interaction with patients in the hospital facility at Kalaupapa. Students expressed a desire to gain an understanding of rising above diversity through personal testimonies of the patients. Students also noted that it would be constructive to have time set aside to evaluate and share with others the information they acquired.

“Future trips to Kalaupapa could be improved to better facilitate learning by allowing more opportunities to interact with residents and hear their stories. Being able to hear the residents tell their personal stories and experiences is very powerful in helping one to understand the impact of disease.” (Student Reflection #21)

“The only suggestion I could provide, is to include a sharing time… the result is the creation of an even greater bond, and the realization that there is always more to learn about a person.” (Student Reflection #5)

### Gratitude

Respondents expressed gratitude in their reflections. Students described how thankful and honored they were to be afforded this unique experience. Students shared that lessons gained while at Kalaupapa can be taken with them throughout life, and that these lessons will add to their professional development.

“It truly was a blessing to be able to go to Kalaupapa and share this experience with my classmates and the faculty. Getting to learn and see the historical places in Kalaupapa was such a memorable experience…It is also a trip that reminds us to not take anything for granted and helping others is a gift in itself.” (Student Reflection #6)

“In general, my trip to Kalaupapa genuinely changed my outlook on life and provided me with deeper insight into the psychosocial aspects of medicine.” (Student Reflection #3)

## Discussion

### Resilience

Resilience of the patients who were sent to Kalaupapa, as well as the resilience of the students in the program, was a prominent overall theme. Students appeared to appreciate this characteristic demonstrated by the patients. With the loss of identity, separation of family and destruction of social supports, patients relied on their individual resilience to overcome these challenges. Patients also relied on their faith and spirituality in order to find a compelling reason to rebuild and create a new sense of themselves and their community. Through this experiential learning experience, students could appreciate how patients needed to redefine health and disease according to their own terms (LOC1), describe how the lack of services, medical and otherwise, encouraged their own self-reliance (LOC2) and better understand how the loss of identity, family relationships, and familiar social supports impacted society both within and beyond Kalaupapa (LOC4). In a similar way, students reflected on their own personal resilience. Finding the mental and physical strength to persevere allowed each student to discover what is needed not only to achieve success in implementation of the service learning project but also to achieve their goals to become future physicians. Similar to the patients from Kalaupapa, students overcame personal barriers to their academic journey, despite facing past disappointments. In the competitive field of medicine, resiliency is a significant factor that contributes to success for students from underrepresented and underserved populations and is an important part of professional behaviors in the practice of medicine (LOC5).

### Unity

An overarching theme of unity was highlighted by student learners and the patients/residents of Kalaupapa. Students recognized patients’ need to regain a sense of family and community in their collective isolation, and redefine these concepts in order build a support network (LO1). Their ability to pull together influenced the availability of healthcare services at Kalaupapa, and how they are viewed as a collective by healthcare professionals (LO4). Mirroring the patients of Kalaupapa, students unified through fellowship and teamwork as they accomplished mutual goals for the Kalaupapa service learning project. Encouragement of group work was reinforced by the project’s student fundraising initiatives and student led coordination and planning. The overarching theme of unity among and between the community and student learners are in alignment with other service learning experiences in medical education that promote collaborative partnerships and community-based approaches to team-building [3]. As future physicians, students must work together with colleagues as well as other health professionals as part of a multi-disciplinary team (LO3). The development of strong interprofessional skills is critical not only to the success of the service learning project, but also to the students’ eventual goal of becoming health care leaders (LO5).

### Humanism in medicine

The Kalaupapa service learning project immersed students in the historical and modern day patient experience of those impacted by Hansen’s disease and their lives in Kalaupapa. The combination of academic enhancement through student research and thesis development, along with experiential learning in a community-based setting, created an ideal educational opportunity for students to learn about and reflect upon the elements of humanism in medicine. Following the service learning project, students expressed respect and empathy for the patients’ struggles and strengths, as well as those of their peers. The experience also reinforced students’ commitment to service, particularly to populations in rural and underserved communities. This understanding of the history and impact of Hansen’s disease on patients and families helped students develop empathy and concern for underserved communities (LO2). Learners gained a deeper understanding of the patient experience and also of themselves as future physicians (LO5). The majority of the students who participated in the study come from rural communities, disadvantaged backgrounds and are underrepresented in medicine. These factors may have played a role in the students’ ability to emphasize with patients in this community and impacted the results. Previous studies have identified practical strategies to enhancing curricula related to the humanistic aspects of medical training for medical students, residents, and faculty. Such strategies include positive role modeling, establishing a humanistic learning environment and creating learning objectives directly related to psychosocial issues [8, 26, 27]. Medical student “wholeness” and engagement with patients are essential to the training of caring, humanistic and ethical physicians [28]. The findings of this study demonstrate that a longitudinal year-long service learning experience focused on underserved and rural communities may also be a new and effective teaching method to enhance students’ awareness and perspectives of the essential aspects of humanism in medicine.

### Limitations

Limitations of the study included the number of student reflections included in the analysis and limited prompts for the reflection assignment. There was also a potential bias that existed for the thematic reviewers, since two of the four reviewers were instructors of the service learning project. Because the reflections were graded assignments, this may also have impacted the responses provided by the students. The study is also limited since there is no data available regarding the students’ knowledge and attitudes of humanism in medicine prior to the service learning experience. Student participants in the Kalaupapan service learning project come from disadvantaged backgrounds and the majority of the students originate from rural communities of Hawai’i and the Pacific (i.e. Guam and American Samoa). Ninety-five (95 %) of the students included in the study are of Asian and Pacific Islander descent and 71 % self-identifiy as having English as a Second Language (ESL). Although this study may be highly relevant to educational programs that service students from disadvantaged and underrepresented backgrounds, these student factors may have introduced potential bias and limits the study’s transferability to general post-baccalaureate and medical student populations.

### Future directions

Given feedback obtained from the students, there is consideration to extend the amount of time spent during the service learning project while in Kalaupapa. Pre and post surveys of student knowledge, attitudes, and beliefs regarding humanism in medicine would strengthen the program’s ability to effectively assess the learners’ gains related to aspects of humanism in medicine. The instructors would also like to integrate additional community input to determine learning objectives and development of the service learning project over the course of the year and explore the opportunities to follow up with program graduates to assess whether findings of the study are maintained over time.

## Conclusion

In order to meet the growing needs of rural and underserved communities, training programs must prepare a health care workforce that is aware of and prioritizes humanism in medicine. The Kalaupapa service learning experience, in conjunction with classroom teaching and assignments, contributes to student awareness regarding health and disease perspectives, rural and underserved populations, the role of health professionals, epidemiology and psychosocial issues and professionalism in the medical profession. This project serves as a potential educational model that can be used to enhance exposure to and broaden future health care professionals’ perspectives of humanism in medicine.

## Ethical approval

Ethical approval was granted on March 20, 2014 by the University of Hawai’i (UH) Human Studies Program. The project was determined to be exempt from federal regulations pertaining to the protection of human research participants, regulations code 45CFR 46.101 (b) (Exempt Category 4). IRB: DOC032114-03212014071409.

## Availability of data and materials

The raw data will not be shared because the combination of variables may identify study participants. These participants are students who were previously enrolled in the ‘Imi Ho’ōla Post-Baccalaureate Program.

## Abbreviations

AAMC: Association of American Medical Colleges
JABSOM: John A. Burns School of Medicine
LCME: Liaison Committee on Medical Education
LO1: learning objective 1
LO2: learning objective 2
LO3: learning objective 3
LO4: learning objective 4
LO5: learning objective 5
SBOM: Scientific Basis of Medicine
